# How many data clusters are in the Galaxy data set? Bayesian cluster analysis in action

**DOI:** 10.1007/s11634-021-00461-8

**Published:** 2021-08-26

**Authors:** Bettina Grün, Gertraud Malsiner-Walli, Sylvia Frühwirth-Schnatter

**Affiliations:** Wirtschaftsuniversität Wien, Welthandelsplatz 1, 1020 Wien, Austria

**Keywords:** Bayes, Cluster analysis, Galaxy data set, Mixture model, Prior specification, 62H30, 62F15, 62C10, 62G07

## Abstract

In model-based clustering, the Galaxy data set is often used as a benchmark data set to study the performance of different modeling approaches. Aitkin (Stat Model 1:287–304) compares maximum likelihood and Bayesian analyses of the Galaxy data set and expresses reservations about the Bayesian approach due to the fact that the prior assumptions imposed remain rather obscure while playing a major role in the results obtained and conclusions drawn. The aim of the paper is to address Aitkin’s concerns about the Bayesian approach by shedding light on how the specified priors influence the number of estimated clusters. We perform a sensitivity analysis of different prior specifications for the mixtures of finite mixture model, i.e., the mixture model where a prior on the number of components is included. We use an extensive set of different prior specifications in a full factorial design and assess their impact on the estimated number of clusters for the Galaxy data set. Results highlight the interaction effects of the prior specifications and provide insights into which prior specifications are recommended to obtain a sparse clustering solution. A simulation study with artificial data provides further empirical evidence to support the recommendations. A clear understanding of the impact of the prior specifications removes restraints preventing the use of Bayesian methods due to the complexity of selecting suitable priors. Also, the regularizing properties of the priors may be intentionally exploited to obtain a suitable clustering solution meeting prior expectations and needs of the application.

## Introduction

This paper investigates the impact of different prior specifications on the results obtained in Bayesian cluster analysis based on mixture models. Mixture models may be used to either approximate arbitrary densities in a semi-parametric way or in a model-based clustering context to identify groups in the data. We will focus on the later application where each component is assumed to potentially represent a data cluster and the cluster distribution is not approximated by several mixture components.

Hennig and Liao (2013) claim that “there are no unique ‘true’ or ‘best’ clusters in a data set” but that the prototypical shape of a cluster needs to be specified before this question can be answered. For clustering methods using mixture models, the prototypical shape of a cluster is in general specified by selecting the component-specific distributions. For the fitted mixture model, then a one-to-one relationship between components and clusters is assumed. For example, in the case of multivariate metric data one can specify isotropic Gaussian distributions as component distributions, where the variance is comparable across components, or Gaussian distributions with arbitrary variance-covariance matrices, which are allowed to considerably vary across components (see, for example, Fraley and Raftery 2002).

The Bayesian framework provides a principled approach to specify the prototypical shape of the clusters. By specifying priors on the model parameters, both the mean prototypical shape as well as the variability around this prototypical shape are included, i.e., what the shape on average is as well as how much the component distributions vary across components. In this sense the Bayesian approach provides, compared to other clustering methods, more flexibility to incorporate the prototypical shape of a cluster in the analysis and hence to arrive at a suitable clustering solution for the specific analysis undertaken. In addition the Bayesian framework also allows to specify a prior on the component weights, thus influencing if the clusters are a-priori assumed to be rather balanced in size or if the clustering solution includes both very small and very large clusters. By contrast, for example, *k*-means clustering assumes that the clusters have an isotropic shape with similar cluster size and volume (see, for example, Grün 2019).

However, the additional flexibility provided by the Bayesian approach might also be perceived as overwhelming, in particular, if the influence of different prior specifications on results obtained remains rather opaque. Aitkin (2001) compares maximum likelihood and Bayesian analyses of mixture models and expresses reservations about the Bayesian approach due to the fact that the prior assumptions imposed remain rather obscure while playing a major role in the results obtained and conclusions drawn. Having sufficient insight into the influence of prior specifications on the clustering results is crucial to leverage the advantages of the Bayesian approach where the priors may be used to regularize the problem and also guide the analysis to focus on the clustering solution of interest.

In the following we consider the mixture of finite mixture model (MFM), a name coined by Miller and Harrison (2018) following Richardson and Green (1997), in the generalized form proposed in Frühwirth-Schnatter et al. (2020). The MFM is a hierarchical finite mixture model where a prior on the number of components *K* is included. We focus on the MFM, because the Bayesian analysis of the MFM results in an a-posteriori distribution of the number of data clusters $K_+$ as well as an a-posteriori distribution of partitions $\mathcal {C}$. These are both core components of a Bayesian cluster analysis to address the questions how many data clusters there are in the data set and how the observations should be grouped into these data clusters.

Note that in our analyses of the MFM, we make a crucial distinction between *K*, the number of components in the mixture distribution, and $K_+$, the number of *filled* components, to which observations are actually assigned. Only a filled component corresponds to a data cluster. This implies that, when estimating the number of clusters in the data, the posterior of $K_+$ is of interest, rather than the posterior of *K*. Previously, Nobile (2004) already differentiated between *K* and $K_+$ when analyzing finite mixture distributions. Also McCullagh and Yang (2008) made the distinction between clusters in the population (*K*) and clusters in the observed sample ($K_+$) and noted that usually a data set contains little information about the clusters in the population, while being more informative regarding the number of clusters in the data set. We will thus not only investigate the prior on *K*, but also explicitly inspect the prior on $K_+$, which is induced by the prior on *K* and the prior on the mixture weights. In the analysis of the results focus is given to the posterior of $K_+$ (rather than *K*), determining in particular the mode of this distribution and its entropy.

We illustrate the impact of different prior specifications using a MFM of univariate Gaussian distributions for the (in-)famous Galaxy data set originally introduced to the statistical literature by Roeder (1990). Several results obtained for this data set using either maximum likelihood estimation or Bayesian analysis methods were compared and discussed in Aitkin (2001). Aitkin (2001) concluded that the maximum likelihood analysis, while having complications of its own, would be rather straightforward to implement and be well understood. By contrast, Aitkin (2001) formulated a call for action with respect to the Bayesian analysis, asking for a careful analysis of the role of the priors. This paper aims at responding to this call for action. Results for the Galaxy data set are complemented with results of a simulation study with artificial data to provide further empirical evidence to arrive at recommendations for suitable prior specifications to obtain a meaningful clustering result.

## Model specification

In our specification of the MFM model with Gaussian component distributions, the following data generation process is assumed for a univariate data set of size *n* given by $\varvec{y} = (y_1,\ldots , y_n)$ (see also Richardson and Green 1997). One assumes that the number of components *K* of the mixture model is sampled from the prior *p*(*K*). Given *K* the component weights $\varvec{\eta } = (\eta _1, \ldots ,\eta _K)$ are sampled from a symmetric *K*-dimensional Dirichlet distribution with parameter $\gamma _K$. For each observation *i* component assignments $S_i$ are drawn from a multinomial distribution with parameter $\varvec{\eta }$.

Regarding the Gaussian component distributions, the component means $\mu _k$ and the component variances $\sigma ^2_k$, $k=1,\ldots ,K$, are independently drawn from the same prior distributions to have exchangeability. The component means $\mu _k$ are drawn from a normal distribution with mean $b_0$ and variance $B_0$, while the component precisions $\sigma ^{-2}_k$, i.e., the inverse variances, are assumed to follow a Gamma distribution with parameters $c_0$ and $C_0$ (and expectation $c_0/C_0$). Note that for the prior for the component distributions not the conjugate prior for the normal distribution with unknown mean and variance is used, but the independence prior is employed. If instead the conjugate prior had been used, the component-specific variances would influence the prior variability of the component means. This would imply that components which have less variability also have their mean closer to the prior mean $b_0$. This prior implication does in general not seem to be appealing in the mixture context and hence the independence prior is used. For a further detailed discussion of the priors for the component distributions see Frühwirth-Schnatter (2006, Chapter 6).

Summarizing, this specification results in the following Bayesian hierarchical MFM model:

1 $$\begin{aligned} \nonumber K&\sim p(K),\\ \nonumber \varvec{\eta }|K&\sim \mathcal {D}_K(\gamma _K),\\ S_i|\varvec{\eta }&\sim \mathcal {M}(\varvec{\eta }), \quad i=1,\ldots ,n,\\ \nonumber \mu _k|b_0,B_0&\sim \mathcal {N}(b_0,B_0), \quad k=1,\ldots K,\\ \nonumber \sigma ^{-2}_k|c_0,C_0&\sim \mathcal {G}(c_0,C_0), \quad k=1,\ldots K,\\ \nonumber y_i|\varvec{\mu },\varvec{\sigma }^2,S_i = k&\sim \mathcal {N}(\mu _k,\sigma ^2_k), \quad i=1,\ldots ,n, \end{aligned}$$

where $$\varvec{\mu } = (\mu _k)_{k = 1,\ldots ,K}$$ and $$\varvec{\sigma }^2 = (\sigma _k^2)_{k = 1,\ldots ,K}$$.

Additionally, hyperpriors might be specified. For example, Richardson and Green (1997) suggest to specify a hyperprior on $C_0$ and Malsiner-Walli et al. (2016) add an additional layer for the prior on the component means which corresponds to a shrinkage prior allowing for variable selection. In the following we do not consider adding hyperpriors in order to be able to assess the influence of different specifications of these priors and their parameters on the clustering results. In this paper we focus on the specification of the following priors and parameters:

- The prior *p*(*K*) of the number of components *K*,
- The value $\gamma _K$ used for the Dirichlet prior,
- The prior parameters $b_0$ and $B_0$ for the component means,
- The prior parameters $c_0$ and $C_0$ for the component variances.

## The Galaxy data set in statistics

The Galaxy data set was originally published in astronomy by Postman et al. (1986) and consists of univariate measurements representing velocities of galaxies, moving away from our galaxy. In this original publication 83 observations are listed. Roeder (1990) introduced the data set to the statistics literature, but omitted the smallest observation such that in the following in the statistics literature only 82 observations were considered. Unfortunately Roeder (1990) also introduced a typo, i.e., one observation has a different value than in Table 1 in Postman et al. (1986). A further influential statistics publication using the Galaxy data set was Richardson and Green (1997) who also considered only 82 observations, but corrected the typo and scaled the units by 1000.

The data set was used in statistics by a number of authors to demonstrate density estimation methods and investigate mixture modeling approaches. They either used the version presented by Roeder (1990) or by Richardson and Green (1997). A number of textbooks on applied statistics also use the data set to demonstrate different statistical methods (see, e.g., Lunn et al. 2012; Hothorn and Everitt 2014).

In the following we will use the Galaxy data set as used by Richardson and Green (1997). This version of the data set was also used by Aitkin (2001) when comparing maximum likelihood and Bayesian analysis methods for estimating mixture models, focusing in particular on the question of the number of data clusters in the data set. Within the maximum likelihood framework, Aitkin (2001) considered mixtures of univariate Gaussian distributions with equal as well as unequal variances. The mixture models were fitted using the EM algorithm (Dempster et al. 1977) and for each class of component distributions, the number of components were selected based on the results of a bootstrap likelihood ratio test (Aitkin et al. 1981; McLachlan 1987). This maximum likelihood analysis may easily be replicated using the R package **mclust** (Scrucca et al. 2016) using also the Bayesian information criterion (BIC) instead of the likelihood ratio test for model selection. Based on the maximum likelihood results, Aitkin (2001) concludes that “there is convincing evidence of three equal variance components, or four unequal variance components, but no convincing evidence of more than these numbers, in the velocity data” (p. 296).

In addition, Aitkin (2001) reviews the Bayesian analysis of the Galaxy data set presented in Escobar and West (1995), Carlin and Chib (1995), Phillips and Smith (1996), Roeder and Wasserman (1997) and Richardson and Green (1997). Table 3 in Aitkin (2001), according to its caption, summarizes the posterior distributions of *K*. However, in fact for the Dirichlet process mixture fitted by Escobar and West (1995), the posterior distribution of $K_+$ is given. The Bayesian approaches compared differ considerably with respect to the prior on *K* and the prior on the component-specific variances and lead to rather diverse results. Aitkin (2001) concludes that some of the Bayesian analysis result in overwhelming posterior evidence for three groups, while other posterior distributions obtained are either relatively diffuse over 4–9 with a mode around 6–7 or are concentrated on the range 7–9. Overall the cluster solutions for the Galaxy data set are interpreted as either being sparse, with up to four clusters, or contain many, i.e., more than four, clusters.

## Prior specifications

In this section, we discuss possible specifications and previous suggestions in the literature for each of the prior distributions and their parameters, taking in particular those into account considered in the Bayesian analysis reviewed in Aitkin (2001). We also discuss our expectation regarding the effect of these prior specifications on the cluster solutions obtained, focusing in particular on the estimated number of data clusters.

### Prior on *K*

Frühwirth-Schnatter et al. (2020) provide an overview on previously used priors on *K* including the uniform distribution (Richardson and Green 1997), the truncated Poisson distribution (Phillips and Smith 1996; Nobile 2004) and the shifted geometric distribution (Miller and Harrison 2018). They also propose the shifted beta-negative-binomial (BNB) distribution as a suitable alternative which represents a generalization of the Poisson and the geometric distribution.

Based on this overview, we consider the following priors on *K*:

- The uniform distribution $K \sim \text {U}(1, 30)$ with prior mean $\mathbb {E}[K] = 15.5$ and prior variance $\mathbb {V}[K] = 74.9$ (Richardson and Green 1997),
- The truncated Poisson distribution $K \sim \text {trPois}(3)$ with prior mean $\mathbb {E}[K] = 3.2$ and prior variance $\mathbb {V}[K] = 2.7$ (Phillips and Smith 1996),
- The shifted geometric distribution $K-1\sim \text {Geom}(0.1)$ with prior mean $\mathbb {E}[K] = 10$ and prior variance $\mathbb {V}[K] = 90$ (Miller and Harrison 2018),
- The shifted BNB distribution $K-1\sim \text {BNB}(1, 4, 3)$ with prior mean $\mathbb {E}[K] = 2$ and prior variance $\mathbb {V}[K] = 4$ (Frühwirth-Schnatter et al. 2020).

These priors essentially cover all Bayesian MFM analysis reviewed and compared by Aitkin (2001). The only exceptions are Carlin and Chib (1995) who perform model selection to decide between a 3- and a 4-component solution and Roeder and Wasserman (1997) who use a uniform distribution with support $\{1, 2, \ldots , 10\}$. Richardson and Green (1997) point out that the upper bound of 30 for the uniform distribution is inconsequential for their applications, including the Galaxy data set, because this bound is never hit during sampling from the posterior distribution. We thus also use this uniform prior for the Galaxy data set.

The proposed priors for *K* differ considerably in the prior means and variances induced. The shifted $\text {BNB}(1, 4, 3)$ has the smallest prior mean; the truncated Poisson distribution has the smallest prior variance, with only a slightly higher prior mean. We expect the two prior distributions $\text {trPois}(3)$ and the shifted $\text {BNB}(1, 4, 3)$, which have comparable, small means, to induce cluster solutions with less data clusters compared to the other two priors. We expect this behavior to be most pronounced for the truncated Poisson distribution, because of its lowest variance, thus putting only very little mass on large values of *K*, e.g., the probability of $K > 10$ is less than 0.001.

### Prior parameter $\gamma _K$ for the component weights

All Bayesian MFM analyses considered in Aitkin (2001) are based on a MFM with $\gamma _K \equiv 1$. However, as will be demonstrated in Sect. 4.3, the Dirichlet parameter $\gamma _K$ crucially affects the prior on $K_+$, since it determines how closely the prior on $K_+$ follows the prior on *K*. A more detailed discussion on the specification of $\gamma _K$ for the MFM is given in Frühwirth-Schnatter et al. (2020).

Frühwirth-Schnatter et al. (2020) suggest to use an arbitrary sequence for the Dirichlet parameter $\gamma _K$ which might depend on the number of components *K*. They distinguish two special cases: the static MFM where $\gamma _K \equiv \gamma$ and the dynamic MFM where $\gamma _K = \alpha / K$. McCullagh and Yang (2008) already discussed these two special cases indicating that they are structurally different. While previous applications of the MFM focused on the static case, the Dirichlet process mixture model is included in the dynamic case.

In the following we will consider the static as well as the dynamic MFM, with $\gamma \in \{0.01, 1, 10\}$ in the static case and $\alpha \in \{0.01, 1, 10\}$ in the dynamic case. Thus, in addition to the popular choice $\gamma \equiv 1$, we consider also a much smaller value of $\gamma$ and $\alpha$ as well as a much larger value. The much smaller value is expected to induce a sparse cluster solution with only very few data clusters and thus also achieve a certain independence of the specification of the prior on *K*. The much larger value is expected to induce cluster solutions with rather equally sized data clusters and also a stronger link between the number of data clusters and the number of components, which implies a larger influence of the prior on *K* in this setting. We expect that the dynamic MFM leads to sparser solutions than the static MFM given that $\gamma _K =\alpha /K$ is likely to assume small values for large *K*.

### Induced prior of the number of data clusters $K_+$

As investigating the posterior of $K_+$, the number of filled components, is the aim of the analysis, it is illuminating to study the prior on $K_+$. The prior on $K_+$ is implicitly induced through the specification of the prior on *K* and the prior parameter $\gamma _K$. Frühwirth-Schnatter et al. (2020) and Greve et al. (2020) present formulas to derive this implicit prior in a computational efficient way. We investigate the prior on $K_+$ induced by the prior specifications on *K* and $\gamma _K$ considered for the Galaxy data set to further gauge our prior expectations of the influence of these prior specifications on the cluster solutions obtained.

**Fig. 1:**
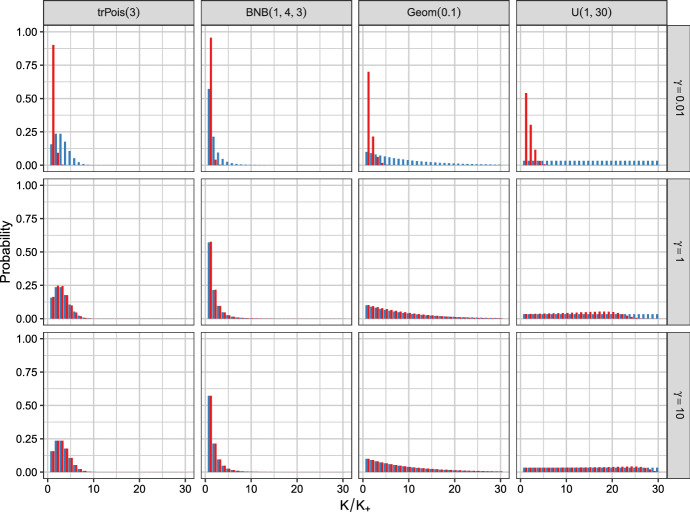
The prior probabilities of *K* (in blue) and $K_+$ (in red) for the static MFM for different priors on *K* and values for $\gamma$ with $n = 82$

**Fig. 2:**
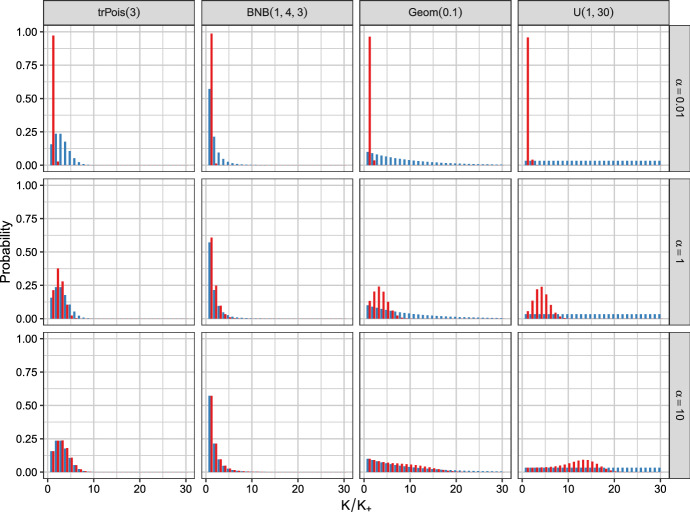
The prior probabilities of *K* (in blue) and $K_+$ (in red) for the dynamic MFM for different priors on *K* and values for $\alpha$ with $n = 82$

Using $n = 82$ – the sample size of the Galaxy data set – the priors on *K* (in blue) and on $K_+$ (in red) are visualized by bar plots in Fig. 1 for the static MFM and in Fig. 2 for the dynamic MFM. The different priors on *K* are in the columns and the values $\gamma \in \{0.01, 1, 10\}$ and $\alpha \in \{0.01, 1, 10\}$ are in the rows. The priors on *K* are ordered according to the mean of $K^2$, i.e., the squared mean of *K* plus the variance of *K*. These specifications on $(K, \gamma _K)$ result in 12 combinations in total inducing different priors on the data clusters $K_{+}$ for the static as well as the dynamic MFM. Comparing Fig. 1 with Fig. 2 indicates that in general the dynamic MFM leads to priors on $K_+$ inducing stochastically smaller values.

Figure 1 clearly indicates that only $\gamma = 0.01$ leads to a sparse prior on the number of data clusters $K_+$ and that the impact of the prior on *K* increases with increasing $\gamma$. For $\gamma = 10$, the two priors *p*(*K*) and $p(K_+)$ are essentially the same. For the dynamic case shown in Fig. 2, the prior on the number of data clusters $K_+$ induces a very sparse solution for $\alpha = 0.01$ regardless of the prior on *K*. For $\alpha = 1$, the prior on $K_+$ is sparser than the prior on *K* but the induced prior clearly considerably varies depending on the selected prior on *K*. For $\alpha = 10$ a close link between the priors on *K* and $K_+$ is discernible if the prior on *K* puts essentially all mass on small values of *K*, while still considerable differences between these two priors are visible for the shifted geometric prior and the uniform prior on *K* which assign substantial mass to values $K > 10$.

In summary, if a sparse clustering solution is of interest, also a sparse prior on $K_+$ should be specified. This can be achieved by specifying a sparse prior on *K* and/or small values for $\gamma /\alpha$. In contrast a flat prior on *K* (e.g., $\text {U}(1, 30)$) and large values of $\gamma /\alpha$ will a-priori support large values of $K_+$ (i.e., larger than 4).

### Prior parameters $b_0$ and $B_0$ for the component means

Richardson and Green (1997) proposed to use empirical Bayes estimates for $b_0$ and $B_0$ which correspond to the midpoint of the observed data range for $b_0$ and the squared length of the observed data range $R^2$ for $B_0$. This choice makes the prior invariant to the scaling of the data, i.e., invariant to the units of the data used or standardization of the data. Richardson and Green (1997) argue that this is a sensible weakly informative prior which does not constrain the component means and does not encourage mixtures with close component means. They perform a sensitivity analysis for this prior by considering values ranging from $R^2/10^2$ to $R^2$ for $B_0$, indicating for the Acidity data set (Crawford et al. 1992) that the estimated number of components are inverse U-shaped, by first increasing with increasing values for $B_0$ and then decreasing again.

In the following we also use the midpoint of the data for $b_0$. For $B_0$ we vary the values to assess the impact on the estimated number of data clusters by considering the values $B_0 \in \{6.3, 20, 100, 630\}$. The extreme values correspond to the limits $R^2/10^2$ and $R^2$ considered by Richardson and Green (1997), 20 corresponds to the empirical variance of the data and Phillips and Smith (1996) used 100 in their analysis.

**Fig. 3:**
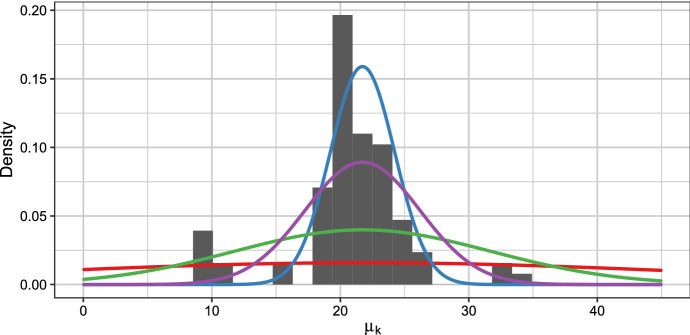
The prior distributions for the component means $\mu _k \sim N(b_0, B_0)$ with $b_0$ equal to the data midpoint and $B_0 \in \{6.3, 20, 100, 630\}$, represented by the blue, purple, green and red line respectively, together with a histogram of the Galaxy data set

Figure 3 visualizes these prior distributions for the component means together with a histogram of the Galaxy data set. $B_0 = R^2 = 630$ induces a flat, weakly informative prior as suggested by Richardson and Green (1997) with approximately the same prior density values assigned to all data values observed. $B_0 = R^2 / 100 = 6.3$ induces the tightest prior for the component means and assigns very low prior density values to the extreme data values, thus shrinking the prior component means towards $b_0$. The smallest value for $B_0$ seems problematic as hardly any weight is assigned to values below 15 or above 30, where, however, the histogram would suggest that the centers of small data clusters are located. We consider this rather extreme range of $B_0$ values to assess whether the inverse U-shape for the estimated number of data clusters can also be observed for the Galaxy data set.

### Prior parameters $c_0$ and $C_0$ for the component variances

Richardson and Green (1997) propose to use $\sigma ^{-2}_k \sim \mathcal {G}(c_0, C_0)$ with a hierarchical prior on $C_0$, but also assess differences in results for a fixed and a random $C_0$. As we are interested in assessing the impact of different prior specifications, we only consider the case of fixed values for $C_0$. Following Escobar and West (1995), Phillips and Smith (1996) and Richardson and Green (1997), we use $c_0 = 2$. We consider $C_0 \in \{0.5, 1, 5, 12.5\}$, where $C_0 = 0.5$ is used in Phillips and Smith (1996), $C_0 = 1$ in Escobar and West (1995), and $C_0 = 12.5$ corresponds to the mean value considered for the random $C_0$ in Richardson and Green (1997).

**Fig. 4:**
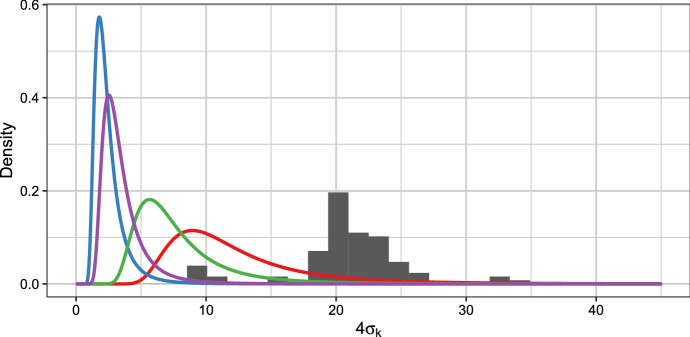
The prior distributions for $4 \sigma _k$ induced by the prior on the component precisions $\sigma _k^{-2} \sim \mathcal {G}(c_0, C_0)$ with $c_0 = 2$ and $C_0 \in \{0.5, 1, 5, 12.5\}$, represented by the blue, purple, green and red line respectively, together with a histogram of the Galaxy data set

Figure 4 visualizes these prior distributions for the component variances together with a histogram of the Galaxy data set. The priors induced for $4\sigma _k$ are visualized. These values correspond to the length of the 95% prediction interval for a single component and might be thus seen as representing the volume considered for the components and hence reflect the prototypical shape imposed for the clusters. Clearly $C_0 = 0.5$ or $C_0 = 1$ induce prior standard deviations which allow to include components able to capture the extreme observations in data clusters of their own, whereas $C_0 = 12.5$ suggests to approximate the data with overlapping component distributions. Small values of $C_0$ induce a fine-grained density approximation, whereas large values of $C_0$ lead to a coarse density approximation and hence we expect the number of estimated data clusters to decrease for increasing $C_0$.

## Posterior inference

In order to obtain samples of the entire parameter vector, which consists of *K* and, conditional on *K*, of $\varvec{\eta } = (\eta _k)_{k = 1,\ldots ,K}$, $\varvec{\mu } = (\mu _k)_{k = 1,\ldots ,K}$, and $\varvec{\sigma }^2 = (\sigma _k^2)_{k = 1,\ldots ,K}$, from the posterior distribution, a transdimensional sampler is required which is able to sample parameter vectors of varying dimension. We use the telescoping sampler proposed by Frühwirth-Schnatter et al. (2020). This MCMC sampling scheme includes a sampling step where *K* is explicitly sampled as an unknown parameter, but otherwise requires only sampling steps used for finite mixtures.

The posterior inference uses data augmentation and also samples the component assignments $\varvec{S} = (S_i)_{i=1,\ldots ,n}$. These latent component assignments induce random partitions of the data. Thus the sampling scheme also allows to directly obtain the posterior distribution of the partitions $\mathcal {C} = \{\mathcal {C}_1, \ldots , \mathcal {C}_{K_+}\}$ of the data and the induced number of data clusters $K_+$, with $\mathcal {C}_k$ being the index set of observations assigned to the *k*th group of the partition $\mathcal {C}$. To illustrate the connection between the component assignments $\varvec{S}$ and the partitions, assume that $K=3$ and $\varvec{S} = (2, 1, 1, 2, 1, 2, 1, 1, 1, 1)$ for $n = 10$ observations. Then $K_+ = 2$, since no observations are assigned to the third component, and the induced partition is given by $\mathcal {C} = \{\mathcal {C}_1, \mathcal {C}_2\}$ with $\mathcal {C}_1 = \{2, 3, 5, 7, 8, 9, 10\}$ and $\mathcal {C}_2 = \{1, 4, 6\}$.

Following Frühwirth-Schnatter et al. (2020), the sampling steps of the telescoping sampler consist of:

1. Update the partition $\mathcal {C}$ by sampling $\varvec{S}$ from $p(\varvec{S} | \varvec{\eta }, \varvec{\mu }, \varvec{\sigma }^2, \varvec{y})$ given by
  $$\begin{aligned} P(S_i=k | \varvec{\eta }, \varvec{\mu }, \varvec{\sigma }^2, y_i) \propto \eta _k f_N(y_i|\mu _k,\sigma ^2_k). \end{aligned}$$
  Determine $N_k=\#\{i=1,\ldots ,n|S_i=k\}$ for $k=1, \ldots ,K$, i.e., the number of observations assigned to $\mathcal {C}_k$, the *k*th group in the partition $\mathcal {C}$ and the number $K_+ = \sum _{k=1}^K I\{N_k>0\}$ of non-empty components with $I\{\cdot \}$ the indicator function. Relabel the components such that the first $K_+$ components are non-empty.
2. Conditional on $\mathcal {C}$, update the parameters of the non-empty components for $k=1,\ldots , K_+$:
  - Draw the component-specific precisions from the posterior:
    $$\begin{aligned} \sigma _k^{-2} | \mu _k, \mathcal {C}, \varvec{y}&\sim \mathcal {G}(c_k, C_k), \end{aligned}$$
    with
    $$\begin{aligned} c_k&= c_0 + \frac{N_k}{2},&C_k&= C_0 + \frac{1}{2} \sum _{i \in \mathcal {C}_k} (y_i - \mu _k)^2. \end{aligned}$$
  - Draw the component-specific means from the posterior:
    $$\begin{aligned} \mu _k | \sigma ^{-2}_k, \mathcal {C}, \varvec{y}&\sim \mathcal {N}(b_k, B_k), \end{aligned}$$
    with
    $$\begin{aligned} b_k&= B_k (B_0^{-1}b_0 + \sigma _k^{-2} N_k\bar{y}_k),&B_k&= (B_0^{-1} + N_k \sigma _k^{-2})^{-1}, \end{aligned}$$
    where $\bar{y}_k$ is the sample mean of the observations assigned to $\mathcal {C}_k$.
3. Conditional on $\mathcal {C}$, draw a new value of *K* using
  $$\begin{aligned} p(K|\mathcal {C})&\propto p(\mathcal {C}|K)p(K)\propto \frac{K!}{(K-K_+)!} \frac{\Gamma (K\gamma _K)}{\Gamma (K \gamma _K +N)} \prod _{k=1}^{K_+} \frac{\Gamma (N_k+ \gamma _K)}{\Gamma (1+ \gamma _K)} p(K). \end{aligned}$$
4. Add $K-K_+$ empty components with component-specific parameters drawn from the priors:
  $$\begin{aligned} \mu _k&\sim \mathcal {N}(b_0, B_0),&\sigma _k^{-2}&\sim \mathcal {G}(c_0, C_0), \end{aligned}$$
  for $$k=K_+ + 1, \ldots , K$$.
5. Conditional on $\varvec{N} = (N_1, \ldots , N_{K_+}, \varvec{0}_{K - K_+})$, with $\varvec{0}_{K - K_+}$ being a $K-K_+$ vector of zeros, draw a new value of $\varvec{\eta }$:
  $$\begin{aligned} \varvec{\eta } | \varvec{N}&\sim \mathcal {D}_K(\varvec{\gamma }), \end{aligned}$$
  with $$\varvec{\gamma } = (\gamma _k)_{k=1,\ldots ,K}$$ and
  $$\begin{aligned} \gamma _k&= \gamma _K + N_k. \end{aligned}$$

Inspecting the details of the sampling scheme provides insights into how the prior specifications influence the conditional posterior distributions.

The prior specifications of the component-specific parameters influence Steps 2 and 4. In Step 2, the updates for $c_k$ indicate that $2 c_0$ might be interpreted as a prior sample size and $C_0/c_0$ corresponds to the variance assumed for these prior observations. The choice of $c_0 = 2$ thus corresponds to adding 4 observations a-priori to each component with a variance of $C_0 / 2$. If $C_0 / 2$ is larger than the empirical within-cluster variance, then $C_k$ is increased leading to the sampling of inflated $\sigma ^2_k$ values. This in turn induces more overlap across the component densities and thus potentially leads to a sparser clustering solution with less data clusters estimated.

The updates for $b_k$ indicate that $b_k$ results as a weighted mean of the prior value $b_0$ and the mean of the observations currently assigned to the cluster. According to the formula for $B_k$, the influence of $B_0$ decreases for data clusters containing many observations, as the second summand increases with $N_k$. It is also clear that there is an interaction with the estimate for the component-specific variance, with larger variances allowing the component-specific means to vary more in the posterior updates. For the largest values of $B_0$ considered, we expect that the prior influence is negligible, and that the posterior updates are only influenced by the data points currently assigned to this cluster.

Step 3 is influenced by the choice of the prior on *K* and $\gamma _K$. More details on this step are given in Frühwirth-Schnatter et al. (2020). The new *K* is sampled from a discrete distribution with support $K \ge K_+$. This distribution is the more spread out the more the prior on *K* puts mass on larger values of *K* and the smaller $\gamma _K$ is. In addition the distribution depends on $K_+$ and the data cluster sizes $(N_1,\ldots , N_{K_+})$. This step allows for the birth and death of empty components.

In Step 4 the parameters of the component-specific distributions of the empty components are drawn from the priors. “Unattractive” empty components result in particular when $B_0$ is large and $C_0$ is small. In this case the sampled $\mu _k$ can be located far away from the data and the probability that observations are assigned to this empty component is extremely small in the following Step 1. Thus, the “attractiveness” of the empty components influences whether new empty components are filled and thus, whether the number of filled components increases.

Step 5 is influenced by the choice of $\gamma _K$. In particular for empty components, the value of the Dirichlet parameter only depends on this prior value, influencing the value $\eta _k$ drawn for these components and hence also the probability of such an empty component having observations assigned in Step 1. The smaller $\gamma _k$, the smaller the sampled $\eta _k$ and thus the smaller the probability that an observation will be assigned to this component in Step 1. Furthermore, it can be seen that the prior sample size is equal to $K\gamma _K$. Thus, for the dynamic MFM where $\gamma _K = \alpha / K$ the prior sample size is constant over mixtures with different number of components, whereas for the static MFM where $\gamma _K \equiv \gamma$ the prior sample size linearly increases with the number of components.

## Assessing the impact of different prior specifications for the Galaxy data set

After discussing in detail how the prior specifications might affect the posterior of the number of data clusters, the following analysis investigates whether these theoretical considerations can be empirically verified for the Galaxy data set. The MFM model is fitted to the Galaxy data set with 384 different prior settings, using four different specifications of the prior on *K*, using either the static or the dynamic MFM, considering three different values for the Dirichlet parameter and four different parameters each for $B_0$ and $C_0$ in a full factorial design.

### MCMC estimation

For each prior setting, posterior inference is performed based on 200,000 iterations after 10,000 burn-in iterations with every fourth draw being recorded (i.e., a thinning of four). Initially 10 components are filled. The MCMC algorithm is initialized by specifying values for the component weights and the component-specific parameters. Equal component weights are specified and all component-specific variances $\sigma ^2_k$, $k=1,\ldots ,10$ are set equal to $C_0/2$. The component-specific means $\mu _k$ are set equal to the centroids obtained when applying the *k*-means algorithm to extract 10 clusters from the data set. The MCMC iterations start with Step 1 by assigning observations to the 10 components according to their a-posteriori probabilities.

Partitions are label-invariant. Hence also the number of data clusters or filled components is a label-invariant quantity and it is not necessary to resolve the label switching problem (Redner and Walker 1984) for the following analysis of the results.

### Analysis of results

The analysis of the results focuses on the impact of the prior specifications on the posterior $p(K_+|\varvec{y})$ of the number of data clusters. The mode of $p(K_+|\varvec{y})$ is used as point estimator. In addition, the entropy of the posterior of $K_+$ is determined to indicate how informative this posterior is for a point estimate of $K_+$. The entropy of a discrete random variable *X* with possible outcomes $x_1,\ldots , x_I$ is given by $- \sum _{i=1}^I P(X = x_i) \log (P(X = x_i) )$. Thus, a high entropy value for the posterior of $K_+$ indicates rather equal posterior probabilities for the different values of $K_+$, while a low entropy value results if the posterior is concentrated on a few values.

The marginal impact of each of the prior specifications on the estimated number of data clusters $K_+$, based on the posterior mode, is assessed by averaging the results across all other prior settings. Table 1 shows the corresponding results. On average, the estimated number of data clusters $K_+$ (a) is higher for the static than the dynamic MFM, (b) increases for increasing values of the Dirichlet parameter, (c) is lowest for the truncated Poisson prior followed by the BNB(1, 4, 3) prior and, after a substantial gap, followed by the $\text {Geom}(0.1)$ and finally the uniform $\text {U}(1, 30)$ prior. For the priors on the component-specific parameters, a non-monotonic influence is indicated for $B_0$. The average number of estimated data clusters $K_+$ is highest for $B_0 = 20$, comparable in-between results are obtained for $B_0 = 6.3$ and $B_0 = 100$, and a substantial lower average number of data clusters $K_+$ is estimated for $B_0 = 630$. The influence of $C_0$ on the average number of data clusters estimated is monotonic and the number substantially decreases for increasing values of $C_0$. The marginal effects observed in Table 1 are in line with our prior expectations based on theoretic considerations and previous results.

**Table 1:** Galaxy data set. Average number of estimated data clusters $K_+$, based on the mode, marginally for each of the different prior specifications

MFM	$\hat{K}_+$	$\gamma$ / $\alpha$	$\hat{K}_+$	*p*(*K*)	$\hat{K}_+$	$B_0$	$\hat{K}_+$	$C_0$	$\hat{K}_+$
Static	5.89	0.01	2.98	trPois(3)	3.99	6.3	5.39	0.5	6.93
Dynamic	4.70	1	5.56	$\text {BNB}(1, 4, 3)$	4.35	20	6.69	1	6.21
		10	7.33	Geom(0.1)	6.00	100	5.20	5	4.53
				U(1, 30)	6.82	630	3.90	12.5	3.50

**Fig. 5:**
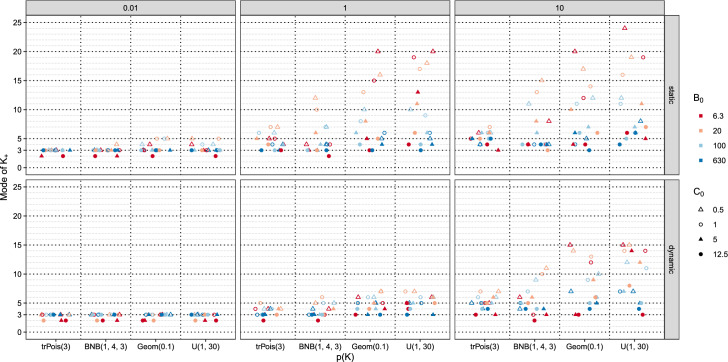
Galaxy data set. Estimated number of data clusters $K_+$, based on the mode, for different prior specifications. In the rows, the results for the static and dynamic MFM are reported, in the columns for $\gamma$ or $\alpha \in \{0.01,1,10\}$, respectively

Figure 5 visualizes the results obtained for the 384 different settings in more detail. This figure allows not only to assess marginal effects, but also to gain insights into the interaction between the prior specifications. For each prior setting, the number of data clusters $K_+$ estimated based on the posterior mode is indicated by a dot with the value being shown on the *y*-axis. The results are split into six panels where the top panels contain the results for the static MFM, while the bottom panels contain the results for the dynamic MFM. The columns represent the different values selected for the Dirichlet parameter, $\alpha$ for the dynamic MFM and $\gamma$ for the static MFM, with values 0.01, 1, and 10 (from left to right). Within each of the panels the results are grouped on the *x*-axis by the prior *p*(*K*). The priors *p*(*K*) are ordered by their prior mean of $K^2$. Colors and point characters are used to indicate the different settings used for the component-specific parameters. Small values of $B_0$ are in red, large values of $B_0$ are in blue. The highly saturated colors indicate the extreme values of $B_0$ and lighter colors are used for the middle values of $B_0$. Filled shapes represent the large values of $C_0$, empty shapes are used for the small values of $C_0$.

Focusing on the dynamic MFM with $\alpha = 0.01$ (in the bottom left panel), one can clearly see that for nearly all settings the number of data clusters $K_+$ are estimated to be equal to 3. Only for some cases, an even smaller number of data clusters $K_+ = 2$ is estimated. This only occurs for settings where $B_0$ is small and $C_0$ is large. This suggests that in this panel, where the dynamic MFM with a sparsity inducing parameter $\alpha$ is fitted, a sparse clustering solution is obtained regardless of prior on *K* and also quite unaffected by the specification on the component-specific parameters.

The results for the static MFM with $\gamma = 0.01$ are shown above this panel (in the top left panel). Clearly the sparsity inducing prior used for $K_+$ leads to the number of data clusters being estimated as equal to three for most settings. Only for very few settings, a lower or a higher number of data clusters than 3 (i.e., 2, 4, or 5) is estimated. Again a lower number of data clusters is only observed in the case where $B_0$ is small and $C_0$ is large. The higher number of data clusters is observed for small values of $C_0$ and middle values of $B_0$.

Overall the results for $\alpha = 0.01$ for the dynamic MFM and $\gamma = 0.01$ for the static MFM indicate that the prior on *K* is not very influential, as regardless of the choice of the prior on *K* a sparsity inducing prior for $K_+$ is imposed where a rather large gap between *K* and $K_+$ a-priori is likely to occur. Also the results are quite insensitive to the selection of the parameters for the component-specific distributions. This implies that if a sparse clustering solution is desired, one clearly needs to use a small value for the Dirichlet parameter. The results are rather insensitive to the specification of the other priors. If the cluster analysis aims at answering the question what is the minimum number of data clusters necessary to approximate the data distribution reasonably well, such a sparsity inducing prior is warranted. In this case the question how many data clusters are in the Galaxy data set would also be rather unambiguously answered by three.

Increasing $\alpha$ and $\gamma$ to 1 indicates that the influence of the other prior specifications on the estimated number of data clusters increases (middle panels). The dynamic MFM tends to estimate less data clusters than the static MFM. The difference to the static MFM becomes more pronounced if the prior on *K* puts more mass on the tails. For the dynamic MFM, all estimated number of data clusters are at most 7, with higher numbers being more likely for the uniform and the geometric prior, followed by the BNB prior and the truncated Poisson prior. Under the static MFM extremely large values are obtained for the uniform and the geometric prior, with estimates as large as 20. These large values are obtained if small values are used in the prior specification for $B_0$ and $C_0$.

For the dynamic MFM, a higher number of data clusters $K_+$ is estimated for $\alpha = 10$ compared to $\alpha = 1$, while for the static MFM, rather similar results are obtained for $\gamma = 1$ and $\gamma = 10$ (panels on the right). For the uniform and geometric prior on *K* the estimated number of data clusters varies most, regardless of whether a static or dynamic MFM is fitted. The prior on *K* is not particularly sparsity inducing and thus the prior on the component-specific parameters influences which approximation of the data density is selected. Small values for $B_0$ induce the most extreme values for the estimated number of data clusters, with large values of $C_0$ leading to small numbers and small values of $C_0$ encouraging large numbers of data clusters.

**Fig. 6:**
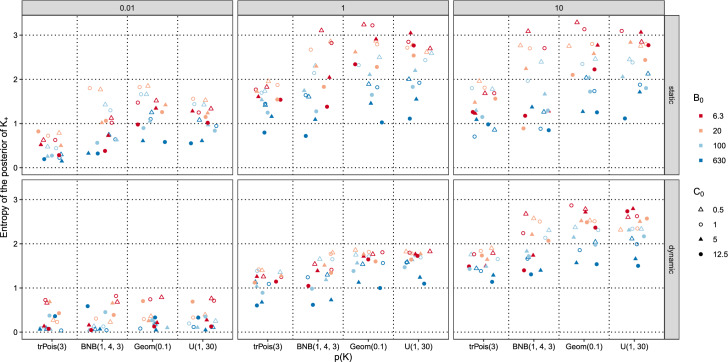
Galaxy data set. Entropy of the posterior of $K_+$ for different prior specifications. In the rows, the results for the static and dynamic MFM are reported, in the columns for $\gamma$ or $\alpha \in \{0.01,1,10\}$, respectively

Figure 6 visualizes the results obtained for the 384 settings in detail based on the entropy of the posterior of $K_+$. If the entropy is 0, then all mass is assigned to a single value (which then also corresponds to the mode shown in Fig. 5). For a fixed support, the uniform distribution has the maximum entropy. For $\text {U}(1, 30)$, the entropy is $\log (30) \approx 3.40$, which corresponds to the case where the posterior of $K_+$ assigns the same probability to each value of $K_+$ from one up to 30.

Figure 6 shows that the entropy values are smallest for the dynamic MFM with $\alpha = 0.01$ with slightly larger values for the static MFM with $\gamma = 0.01$. For the dynamic MFM, the entropy increases for increasing $\alpha$. For the static MFM, the entropy values also increase from $\gamma = 0.01$ to $\gamma = 1$, but are rather comparable for $\gamma = 1$ and $\gamma = 10$.

Regarding the prior on *K*, smaller entropy values are observed for the truncated Poisson prior compared to the other priors which have rather comparable entropy values for a given $\gamma _K$ setting. This indicates that the smaller prior variance of the prior on *K* has a substantial impact on the entropy.

Regarding the prior on $B_0$, a general pattern of the red points being above the blue points is discernible. This implies that the posterior on $K_+$ is particularly spread out for small values of $B_0$, i.e., where the component-specific mean values are shrunken towards the midpoint. We conjecture that in this setting posterior mass is also assigned to small values of $K_+$ as due to the shrinkage there is posterior support for solutions with few data clusters. For example, the observations in the Galaxy data set with large values which seem to form a small data cluster of their own, might be merged with observations from the middle bulk of the observations due to shrinkage, inducing a large component-specific variance and thus a coarse density approximation.

Regarding $C_0$, the general pattern is that the filled shapes are below the empty shapes, indicating that the entropy increases with decreasing values of $C_0$. This means that the probability mass is more spread out if one aims at a fine-grained approximation using a rather small volume as prototypical shape for the clusters. In particular, if the aim is semi-parametric density estimation and a small volume is imposed, it is not to be expected that a single mixture with a specific value of $K_+$ approximates the data distribution well, but rather a range of mixtures with different values of $K_+$ perform well and all well fitting mixtures may be combined to obtain a good approximation.

## Assessing the impact of different prior specifications for artificial data

To complement the results obtained for the Galaxy data set, a simulation study with artificial data is performed where the data generating process and the true number of data clusters are known. Results are obtained and compared for maximum likelihood estimation as well as Bayesian inference with different prior specifications. In the simulation study also the impact of different sample sizes and of fitting a misspecified mixture model is assessed.

### Data generation and analysis setup

We designed the data generating process in the simulation study to induce data sets which are similar to the Galaxy data set. The underlying data generating process is either a mixture of univariate Gaussian distributions or a mixture of univariate uniform distributions with four components each. Two different sample sizes with $n = 100$ and 1000 data points are considered. For $n = 100$, the four cluster sizes are fixed to 5, 55, 30 and 10 and these cluster sizes are multiplied by 10 for $n = 1000$. For the Gaussian mixture, the four component means and standard deviations are given by $\mu _k \in \{9.5, 20, 24.5, 33\}$ and $\sigma _k \in \{0.25, 1, 1, 0.5\}$, respectively. For the uniform mixture, the lower and upper bounds of the four uniform component distributions are given by $\{(9, 10), (18, 22), (22, 27), (32, 34)\}$. 100 different artificial data sets are drawn and analyzed for each of the scenarios.

Results for maximum likelihood estimation are obtained using the R package **mclust**. The default initialization scheme implemented in the package is used and model choice with regard to *K* is performed using the BIC. Model choice consists in selecting the best model within three modeling approaches for the component variances: (1) equal variances across components, (2) unequal variances across components, (3) the best model according to the BIC among the equal and unequal variance models.

The MFM (as given in (1)) is fitted to each of the 100 artificial data sets of each scenario using essentially the same prior specifications as used for the analysis of the Galaxy data set. We only make two modifications. We restrict the prior specifications to the extreme values for $B_0$ and $C_0$, i.e., $B_0 \in \{6.3, 630\}$ and $C_0 \in \{0.5, 12.5\}$, to obtain a more succinct presentation of the results. Furthermore, a uniform prior $\text {U}(0, 100)$ for *K* instead of a uniform prior $\text {U}(0, 30)$ is specified. Given that larger sample sizes are considered, a larger upper bound for the uniform distribution is selected to ensure that the specific bound selected is still inconsequential. We base the posterior inference for each prior setting on MCMC sampling using 200,000 iterations after discarding 10,000 iterations as burn-in samples and using a thinning of four. The same initialization scheme as for the Galaxy data set is employed.

### Analysis of results

First, we inspect the results obtained using maximum likelihood estimation with the BIC for the three modeling approaches for the different sample sizes and data generating processes. It should be noted that BIC selects the number of components *K* rather than the number of data clusters $K_+$. The estimated number of components are summarized in Table 2 for each setting using the minimum, the 25%, 50% and 75% quantile and the maximum to characterize the distribution of these estimates across the 100 data sets.

**Table 2:** Artificial data, maximum likelihood estimation with the BIC. Results are shown for the three different modeling approaches consisting of equal, unequal and equal as well as unequal variances for the component distributions. The estimated number of components are summarized over 100 data sets by the minimum, the 25%, 50% and 75% quantile and the maximum in square brackets

	*n*	Equal	Unequal	Equal or unequal
Gaussian	100	[4.0, 4.0, 5.0, 5.0, 7.0]	[4.0, 4.0, 4.0, 4.0, 5.0]	[4.0, 4.0, 4.0, 4.0, 5.0]
	1000	[6.0, 7.0, 9.0, 9.0, 12.0]	[4.0, 4.0, 4.0, 4.0, 4.0]	[4.0, 4.0, 4.0, 4.0, 4.0]
Uniform	100	[4.0, 5.0, 5.0, 6.0, 8.0]	[3.0, 4.0, 4.0, 5.0, 7.0]	[3.0, 4.0, 5.0, 5.0, 8.0]
	1000	[7.0, 8.8, 9.0, 9.0, 15.0]	[5.0, 6.0, 7.0, 7.0, 9.0]	[5.0, 6.0, 7.0, 7.0, 9.0]

If the data are drawn from a Gaussian mixture and the larger sample size $n=1000$ is considered, maximum likelihood estimation in combination with BIC always selects four components in case the unequal variance model is specified or the best model among the equal and unequal variance models is selected. Only slightly worse results are obtained for the smaller sample size, $n = 100$, when these modeling approaches are considered. If the equal variance model is enforced, the number of components are correctly selected or slightly overestimated for $n = 100$. For the larger sample size, considering only the equal variance model leads to overestimating the number of components by at least two with a median number of five and up to eight components in addition.

If the mixture model is misspecified, the performance of the maximum likelihood estimation deteriorates. This is expected as the BIC takes goodness-of-fit of the estimated density into account to select a suitable number of components for the mixture distribution. For the smaller sample size, $n = 100$, the number of components are only slightly overestimated regardless of the modeling approach. The estimated number of components increases for the larger sample size, $n = 1000$. In this case, the correct number of components is never selected and there are either at least five or seven components included in the final mixture distribution. The maximum likelihood estimation approach thus performs poorly if the model is misspecified and the sample size is rather large.

**Table 3:** Artificial data, Bayesian estimation. Average number of estimated data clusters $K_+$, based on the mode, marginally for each of the different prior specifications and whether the component distributions are Gaussian or uniform distributions

Gaussian											
*n*	$\hat{K}_+$	MFM	$\hat{K}_+$	$\gamma$ / $\alpha$	$\hat{K}_+$	*p*(*K*)	$\hat{K}_+$	$B_0$	$\hat{K}_+$	$C_0$	$\hat{K}_+$
100	5.12	Static	6.15	0.01	3.97	trPois(3)	4.69	6.3	6.34	0.5	6.65
1000	5.87	Dynamic	4.83	1	5.25	$\text {BNB}(1, 4, 3)$	4.97	630	4.65	12.5	4.34
				10	7.26	Geom(0.1)	5.66				
						U(1, 100)	6.66				
Uniform											
*n*	$\hat{K}_+$	MFM	$\hat{K}_+$	$\gamma$ / $\alpha$	$\hat{K}_+$	*p*(*K*)	$\hat{K}_+$	$B_0$	$\hat{K}_+$	$C_0$	$\hat{K}_+$
100	5.56	Static	7.33	0.01	4.85	trPois(3)	5.57	6.3	7.65	0.5	8.61
1000	7.76	Dynamic	5.99	1	6.85	$\text {BNB}(1, 4, 3)$	6.27	630	5.67	12.5	4.71
				10	8.27	Geom(0.1)	6.84				
						U(1, 100)	7.96				

Table 3 summarizes the results for the Bayesian approach with different prior specifications when using the same artificial data as used for maximum likelihood estimation. Here, we report inference regarding the number of data clusters $K_+$ rather than *K*. The table shows the marginal effects of the different prior specifications on the estimated number of data clusters. The effects are again in line with our expectations and confirm the insights gained for the Galaxy data set. More specifically, it can be observed that the number of estimated data clusters increases for increasing prior mean of *K* and smaller values of $C_0$. In addition it can be noted that the model misspecification leads on average to more data clusters being estimated.

**Fig. 7:**
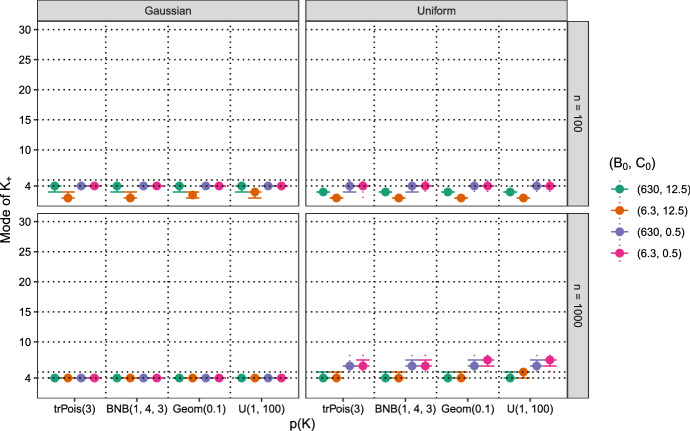
Artificial data, dynamic MFM with $\alpha = 0.01$. Estimated number of data clusters $K_+$ based on the mode for 100 data sets with different prior specifications for the prior on *K*, $B_0$ and $C_0$. In the rows, the results for different samples sizes ($n = 100$ or 1000) are reported, in the columns for different data generating processes, mixtures of Gaussians or mixtures of uniform distributions. The results for the $(B_0, C_0)$ specifications as listed in the legend are shown from left to right within each prior on *K* setting

**Fig. 8:**
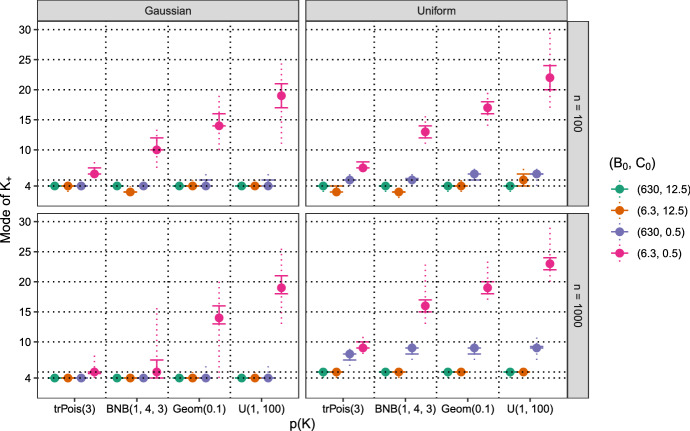
Artificial data, static MFM with $\gamma = 1$. Estimated number of data clusters $K_+$ based on the mode for 100 data sets with different prior specifications for the prior on *K*, $B_0$ and $C_0$. In the rows, the results for different samples sizes ($n = 100$ or 1000) are reported, in the columns for different data generating processes, mixtures of Gaussians or mixtures of uniform distributions. The results for the $(B_0, C_0)$ specifications as listed in the legend are shown from left to right within each prior on *K* setting

In the following the impact of the prior settings on the estimated number of data clusters is investigated in more detail for a dynamic MFM with $\alpha = 0.01$ and a static MFM with $\gamma = 1$. In Figs. 7 and 8, the results over the 100 data sets are summarized. The median estimated number of data clusters $K_+$ is represented by the bullet points. In addition error bars connected by straight lines indicate the range between the 25% and the 75% quantile, whereas dotted lines indicate the total range from minimum to maximum.

Results for the dynamic MFM with $\alpha = 0.01$ shown in Fig. 7 indicate that if the data generating process is a Gaussian mixture, the correct number of data clusters is selected most of the times, in particular if the sample size is large, i.e., for $n = 1000$. For the smaller sample size, $n = 100$, the number of data clusters is underestimated if large variances are a-priori assumed for the component distributions and in particular if also the component-specific means are shrunken together because of the small value of $B_0$. Further, it can also be seen that for the larger sample size the estimated number of data clusters coincide with the true number of data clusters regardless of the prior distributions used for *K* and the other parameters. Thus, if there is no model misspecification and the data set is sufficiently large, using a dynamic MFM with a small $\alpha$ value leads to correct estimates of the number of data clusters regardless of the other prior settings.

If the component distribution is misspecified but the data set is small, the results obtained are rather similar to the Gaussian case. However, for the larger sample size, $n=1000$, four clusters are only estimated if the priors on the component distributions assume large values for $C_0$ and $B_0$. Otherwise, the number of clusters is clearly overestimated with median values between six and seven. Thus, using a dynamic MFM with a small $\alpha$ value in combination with sensible priors on the component distributions results in obtaining the correct estimates for the number of data clusters even if the model is misspecified and the data set is rather large.

The dynamic MFM with a small $\alpha$ value is clearly a successful strategy for obtaining an estimate of the number of data clusters which could be seen as the “minimum number of data clusters” being present in the data. To further emphasize the advantages of this approach, the results for the static MFM with $\gamma = 1$ are, in comparison, inspected in Fig. 8. Regardless of the data generating process and the sample size, using priors on the component distributions which induce small values for $B_0$ and $C_0$ leads to overestimating the number of data clusters in the data set. The amount of this overestimation strongly depends on the prior used for *K*. The estimated number of data clusters in fact increases with the prior mean of *K*, e.g., for $K \sim \text {U}(1,100)$ the median number of estimated data clusters is about 20 regardless of the data generating process and the sample size.

For Gaussian mixtures, using a static MFM with $\gamma =1$ again leads to correct estimates of the number of data clusters for almost all prior specifications. The only exception is the already highlighted setting where the priors on the component distributions induce small values for $B_0$ and $C_0$.

In contrast, for $n = 1000$ the number of data clusters is always overestimated in case of model misspecification and a static MFM with $\gamma = 1$ is fitted. For large values of $C_0$, consistently five data clusters are estimated instead of four. Using a small value for $C_0$ allows for semi-parametric density estimation and hence leads to a substantial overestimation of the number of data clusters. Thus, it is not recommended to use a static MFM with $\gamma =1$ in applications where the component distributions are likely to misspecify the cluster distribution and a sparse clustering solution is of interest.

## Discussion and conclusions

In this paper, we respond to the call for action made by Aitkin (2001) regarding the need to provide more insights into the influence of different prior specifications when fitting Bayesian mixture models. Based on recent developments in the context of MFMs, we use the model specification of a MFM, considering the static as well as the dynamic case. The Galaxy data set is used to illustrate the prior impact on the estimated number of data clusters $K_+$ using the mode as well as on the entropy of the posterior of $K_+$. Results confirm the marginal effects postulated, but also interesting interaction effects are discerned.

Aiming at a sparse clustering solution using a dynamic MFM with $\alpha = 0.01$ gives stable results regardless of the prior on *K*. The clustering solution is also rather insensitive to the prior on the component-specific parameters as long as they are sensible. Such a prior is especially recommended to be combined with large component variances and large variances of the component means, if the data set is large and the cluster density is unknown and likely to be misspecified (which is often the case in applications). Such a prior specification will avoid overfitting and lead to an estimate of $K_+$ that could be interpreted as the “minimum number of data clusters” being present in the data and in general might provide a better clustering performance than the maximum likelihood approach combined with the BIC.

For the Galaxy data set, a dynamic MFM with $\alpha = 0.01$ would lead to an unambiguous estimate of three data clusters with also the posterior distributions being rather concentrated on very few values. This is in line with the conclusion drawn in Aitkin (2001) for the maximum likelihood framework using equal variance components in the mixture model.

We suggest to use the dynamic MFM with small $\alpha$ value and reasonable component-specific distributions in a Bayesian model-based clustering application where a minimum number of data clusters is to be identified. For the component-specific distributions, shrinking the prior mean is not recommended, whereas for the component-specific variances using reasonable values is important to guard against too fine-grained or too coarse approximations. In the univariate case the visualization of the induced volume (see Fig. 4) is useful to determine a suitable value for $C_0$. A generalization of such a visual tool to the multivariate case or other component-specific distributions would be of interest. Further analysis is also required to gain insights of the prior impact on Bayesian cluster analysis results for data sets with many variables and with other component-specific distributions. In addition, if less focus is given to the clustering aspect of the MFM model, it might also be interesting to investigate the posterior of the number of components *K*, in particular based on a simulation study where *K* and $K_+$ are known and may be manipulated to be different.

## References

[CR1] Aitkin M (2001). Likelihood and Bayesian analysis of mixtures. Stat Model.

[CR2] Aitkin M, Anderson D, Hinde J (1981). Statistical modelling of data on teaching styles. J Royal Stat Soc A.

[CR3] Carlin BP, Chib S (1995). Bayesian model choice via Markov chain Monte Carlo methods. J Royal Stat Soc B.

[CR4] Crawford SL, DeGroot MH, Kadane JB, Small MJ (1992). Modeling lake-chemistry distributions: approximate Bayesian methods for estimating a finite-mixture model. Technometrics.

[CR5] Dempster AP, Laird NM, Rubin DB (1977). Maximum likelihood from incomplete data via the EM algorithm. J Royal Stat Soc B.

[CR6] Escobar MD, West M (1995). Bayesian density estimation and inference using mixtures. J Am Stat Assoc.

[CR7] Fraley C, Raftery AE (2002). Model-based clustering, discriminant analysis and density estimation. J Am Stat Assoc.

[CR8] Frühwirth-Schnatter S (2006). Finite mixture and Markov switching models.

[CR9] Frühwirth-Schnatter S, Malsiner-Walli G, Grün B (2020) Generalized mixtures of finite mixtures and telescoping sampling. arXiv:2005.09918

[CR10] Greve J, Grün B, Malsiner-Walli G, Frühwirth-Schnatter S (2020) Spying on the prior of the number of data clusters and the partition distribution in Bayesian cluster analysis. arXiv:2012.12337

[CR11] Grün B (2019) Model-based clustering. In: Frühwirth-Schnatter S, Celeux G, Robert CP (eds) Handbook of mixture analysis. Chapman and Hall/CRC, pp 157–192

[CR12] Hennig C, Liao TF (2013). How to find an appropriate clustering for mixed-type variables with application to socio-economic stratification. J Royal Stat Soc C.

[CR13] Hothorn T, Everitt BS (2014). A handbook of statistical analyses using R.

[CR14] Lunn D, Jackson C, Best N, Thomas A, Spiegelhalter D (2012). The BUGS book: a practical introduction to Bayesian analysis.

[CR15] Malsiner-Walli G, Frühwirth-Schnatter S, Grün B (2016). Model-based clustering based on sparse finite Gaussian mixtures. Stat Comput.

[CR16] McCullagh P, Yang J (2008). How many clusters?. Bayesian Anal.

[CR17] McLachlan GJ (1987). On bootstrapping the likelihood ratio test statistic for the number of components in a normal mixture. J Royal Stat Soc C.

[CR18] Miller JW, Harrison MT (2018). Mixture models with a prior on the number of components. J Am Stat Assoc.

[CR19] Nobile A (2004). On the posterior distribution of the number of components in a finite mixture. The Ann Stat.

[CR20] Phillips DB, Smith AFM, Gilks W, Richardson S, Spiegelhalter DJ (1996). Bayesian model comparison via jump diffusions. Markov Chain Monte Carlo in Practice.

[CR21] Postman M, Huchra JP, Geller MJ (1986). Probes of large-scale structure in the Corona Borealis region. The Astron J.

[CR22] Redner RA, Walker HF (1984). Mixture densities, maximum likelihood and the EM algorithm. SIAM Rev.

[CR23] Richardson S, Green PJ (1997). On Bayesian analysis of mixtures with an unknown number of components. J Royal Stat Soc B.

[CR24] Roeder K (1990). Density estimation with confidence sets exemplified by superclusters and voids in galaxies. J Am Stat Assoc.

[CR25] Roeder K, Wasserman L (1997). Practical Bayesian density estimation using mixtures of normals. J Am Stat Assoc.

[CR26] Scrucca L, Fop M, Murphy TB, Raftery AE (2016). mclust 5: clustering, classification and density estimation using Gaussian finite mixture models. The R J.

